# Associations of the triglyceride-glucose index and atherogenic index of plasma with the severity of new-onset coronary artery disease in different glucose metabolic states

**DOI:** 10.1186/s12933-024-02163-9

**Published:** 2024-02-20

**Authors:** Xiaosheng Wu, Weiping Qiu, Huancheng Yang, Yan-Jun Chen, Jianling Liu, Guojun Zhao

**Affiliations:** 1 Affiliated Qingyuan Hospital, Guangzhou Medical University (Qingyuan People’s Hospital), Qingyuan, Guangdong China; 2 Guangzhou Medical University, Guangzhou, China; 3 Department of Radiology, The Third Affiliated Hospital of Shenzhen University (Luohu Hospital Group), Shenzhen, 518000 China; 4 Shantou University Medical College, Shantou University, Shantou, 515000 China; 5 Department of Pathology, Southwest Hospital, Third Military Medical University, Chongqing, 400038 People’s Republic of China

**Keywords:** Triglyceride glucose index, Atherogenic index of plasma, Coronary artery disease, Coronary artery disease severity, Glucose metabolic states

## Abstract

**Background:**

The triglyceride-glucose (TyG) index is considered a dependable biomarker for gauging insulin resistance. The atherogenic index of plasma (AIP) represents a marker reflecting atherosclerosis. However, there is currently no study specifically exploring the associations of these two biomarkers with the severity of new-onset coronary artery disease (CAD) under different glucose metabolic states. Therefore, this study aims to evaluate the correlations of these two biomarkers with CAD severity in patients newly diagnosed with CAD under various glucose metabolism conditions.

**Method:**

Totally 570 subjects first administered coronary angiography were enrolled, including 431 first diagnosed CAD patients and 139 non-CAD patients. CAD severity  was gauged by the quantity of narrowed arteries (single-vessel and multi-vessel CAD). According to WHO diabetes guidelines, glucose metabolic states were divided into normal glucose regulation (NGR), pre-diabetes mellitus (Pre-DM), and diabetes mellitus (DM). The relationships of the TyG index and AIP with CAD severity were validated by logistic regression analysis, including adjustment for traditional cardiovascular risk elements and medical treatments. Their predictive efficacy for CAD was evaluated by receiver operating characteristic (ROC) curves.

**Result:**

The TyG index and AIP were independently correlated with CAD in accordance with logistic regression analysis (both P < 0.05). Regardless of the glucose metabolic states, there was no statistical correlation between the TyG index and CAD severity. However, AIP in NGR patients was significantly related to CAD severity (P < 0.05). The areas under the curve of the TyG index and AIP for predicting CAD were 0.682 and 0.642 (both P < 0.001), respectively, and their optimal cut-off values were 3.210 (Youden index: 0.305) and 0.095 (Youden index:0.246), respectively.

**Conclusion:**

The TyG index and AIP have significant associations with CAD. The TyG index had no association with CAD severity, regardless of glucose metabolic states. AIP exhibited a discernible link with CAD severity in NGR patients, but not in the pre-DM or DM populations. The TyG index and AIP have similar predictive values for new-onset CAD.

## Introduction

Coronary artery disease (CAD), a chronic cardiac disorder triggered by the narrowing of coronary arteries, represents the top global contributor to mortality [1]. Invasive coronary angiography (CAG), considered the ultimate tool for CAD diagnosis, effectively ascertains both the degree and number of coronary artery stenoses. According to the results of CAG, patients with ≥ 50% lumen constriction in a major coronary artery are diagnosed with CAD [2]. In addition, the quantity of stenotic vessels determines the severity of CAD. Compared with single-vessel CAD, multi-vessel CAD has a relatively lower reperfusion success rate and a higher risk of adverse prognosis in contrast with single-vessel CAD [3]. CAG is invasive and expensive, as well as having a potential risk of serious complications. Many patients refuse to receive CAG examinations during the early stages of CAD, missing timely medical evaluation and coronary revascularization treatment. Moreover, the CAD population has a high recurrence risk, especially those with Type 2 diabetes mellitus (T2DM) [4]. Therefore, timely identification and intervention in patients at high risk of CAD have an important clinical significance.

Insulin resistance (IR) is the major characteristic of T2DM and has been confirmed as a significant driver of CAD [5, 6]. Pathophysiological studies have shown that IR promotes an inflammatory state, vascular endothelial dysfunction, and dyslipidemia, which may be the main mechanisms of CAD progression [7]. The triglyceride-glucose (TyG) index, as a dependable and newfound biomarker for gauging IR, is closely related to the progression of various cardiovascular events [8]. A previous study observed the relation of the TyG index to CAD severity in the prediabetic population [2]. The TyG index is easily available and low-cost, and it is expected to provide valuable data for the clinical management of CAD.

The atherogenic index of plasma (AIP), denoting the logarithmic ratio of TG to high-density lipoprotein cholesterol (HDL-C) in molar concentration, is a parameter for evaluating plasma atherosclerosis and exhibits a profound connection to the atherosclerotic burden and cardiovascular occurrences [9–11]. AIP has been reported to be significantly associated with T2DM [12, 13]. A recent study demonstrated that AIP is positively correlated with the progression from prediabetes to diabetes and is negatively related to the recovery from prediabetes to normoglycemia [14]. The TyG index and AIP are both related to T2DM and cardiovascular events. However, there is currently no study specifically evaluating the role of the TyG index and AIP on new-onset CAD severity under different glucose metabolic states. Therefore, this study aims to validate the associations of the TyG index and AIP with new-onset CAD severity in different states of glucose metabolism.

## Method

### Ethical statements

This study was conducted at the Affiliated Qingyuan Hospital, Guangzhou Medical University (Qingyuan People’s Hospital). The study was implemented in accordance with the Declaration of Helsinki and was authorized by the Ethics Committee of the Affiliated Qingyuan Hospital, Guangzhou Medical University (Qingyuan People’s Hospital) (IRB-2023-003), which waived the requirement for informed consent due to its retrospective nature.

### Study design

**Fig. 1:**
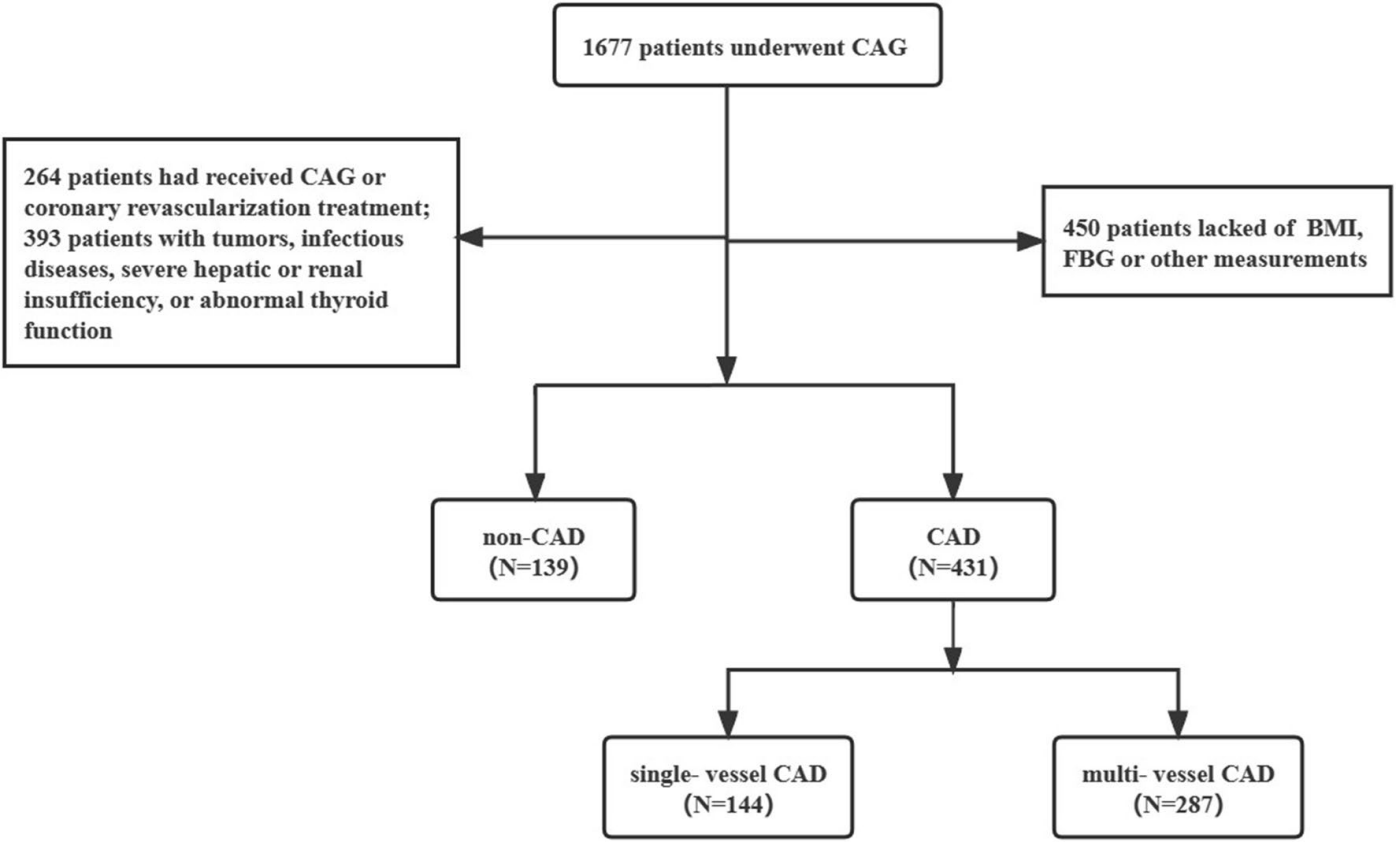
Flow chart of subject recruitment. *CAG* coronary angiography, *CAD* coronary artery disease; BMI, body mass index; FBG, fasting plasma glucose

We scrutinized 1677 patients who underwent their first CAG and were admitted to Affiliated Qingyuan Hospital, Guangzhou Medical University (Qingyuan People’s Hospital) between September 1, 2019 and September 1, 2022. Exclusion conditions were: (1) age below 18 years or above 75 years; (2) previous CAG or coronary revascularization therapy; (3) malignancies, infectious diseases, severe hepatic or renal insufficiency, or impaired thyroid function; and (4) incomplete body mass index (BMI), FBG, or other measurements. Ultimately, 570 subjects were recruited, comprising 431 patients newly diagnosed with CAD and 139 patients without CAD (Fig. 1). In accordance with the established diagnostic criteria for CAD, 570 subjects were classified into the CAD (n = 431) and non-CAD (n = 139) groups. Furthermore, based on the severity of CAD, the CAD group was subdivided into the single-vessel (n = 144) and multi-vessel (n = 287) CAD groups.

### Data source and collection

The data of the patients was collated from the autonomous digital medical record system, including key demographic features, clinical background, outcomes of blood analysis, and relevant medical imaging records. Demographic characteristics comprised age, gender, weight, height, blood pressure, and smoking and alcohol consumption habits. Clinical history encompassed hypertensive and diabetic medical histories as well as the states of medical treatments. Medical treatments embraced antihypertensive, antidiabetic, antilipidemic and antiplatelet drugs.

The blood specimens were acquired in the morning through the collection of fasting venous blood by skilled medical professionals. TG, total cholesterol (TC), HDL-C, low-density lipoprotein cholesterol (LDL-C), FPG, and glycosylated hemoglobin (HbA1c) were assessed on an automated hematology analyzer. Catheter-based invasive CAG was executed, utilizing percutaneous radial or femoral arteriography. The employed angiographic apparatus was furnished with ample versatility, enabling precise diagnoses of all manifestations of coronary arteries.

### Definitions

CAD is characterized by a luminal constriction of ≥ 50% in one principal coronary artery [2]. The severity of CAD is contingent upon the quantity of narrowed coronary arteries. Narrowing of ≥ 50% in the left main artery is also considered multi-vessel CAD.

The AIP is derived from the formula: log10 (TG [mol/L]/HDL-C [mol/L]) [9]. The TyG index is calculated as ln (TG [mg/dL] × FPG [mg/dL]/2) [8].

According to the World Health Organization guidelines on diabetes [15], the diagnostic criteria for diabetes mellitus (DM) include FPG ≥ 7.0 mmol/L, 2-h plasma glucose level ≥ 11.1 mmol/L based on the oral glucose tolerance test, HbA1c ≥ 6.5%, or a history of T2DM. Normal glucose regulation (NGR) is defined as FPG < 6.1 mmol/L and 2-h plasma glucose level < 7.8 mmol/L. Pre-diabetes mellitus (Pre-DM) should be considered for individuals exhibiting elevated plasma glucose levels that do not meet the criteria for a T2DM diagnosis.

### Statistical analysis

Continuous variates were shown as median and interquartile range (IQR), encompassing the 25th (Q25) and 75th (Q75) percentiles. Categorical variables  were presented in the form of counts or percentages. In order to compare continuous variables among various groups, either a one-way analysis of variance or the Kruskal–Wallis test was deployed, while distinctions in categorical variables were compared across the groups using the chi-square test.

Logistic regression models, incorporating odds ratios (ORs) and corresponding 95% confidence intervals (CIs), were established to validate the correlations of the TyG index and AIP (independent variables) with CAD (dependent variable). Within the TyG index quartiles, three models were formulated to scrutinize the interaction of the TyG index with CAD: Model 1, an unadjusted state; Model 2, adjustment by incorporating age and sex as covariates; and Model 3, refinement by introducing the variables from Model 2, in conjunction with systolic blood pressure (SBP), BMI, smoking status, drinking status, HDL-C, LDL-C, hypertension, antihypertensive drugs, antidiabetic drugs, antilipidemic drugs and antiplatelet drugs. Similarly, based on the AIP quartiles, three distinct models were constructed to explore the relationship between AIP and CAD, considering significant covariates: Model 1, an unaltered state; Model 2, inclusion of age and sex; Model 3, further enhancement by the inclusion of variables from Model 2, along with SBP, BMI, smoking status, drinking status, LDL-C, HbA1c, DM, hypertension, antihypertensive drugs, antidiabetic drugs, antilipidemic drugs and antiplatelet drugs.

Additionally, to scrutinize the links between the TyG index or AIP (independent variables) and the severity of newly diagnosed CAD, logistic regression analysis was employed again.

Moreover, the predictive efficacy of these two indexes for CAD entailed the calculation of the area under the curve (AUC) through the receiver operating characteristic (ROC) curve and the computation of the corresponding 95% confidence interval (CI). The best cut-off values refer to the TyG index and AIP corresponding to the maximum Youden index, and the Youden index is equal to the sum of sensitivity and specificity minus 1 [16, 17]. SPSS 26.0 (IBM, USA) and GraphPad Prism 8.0 (GraphPad Software, USA; www.graphpad.com) were utilized for statistical analysis. The threshold of statistical significance was established at P < 0.05.

## Results

### Clinicodemographic features of the non-CAD and CAD groups

Clinicodemographic feature analysis involved 570 subjects, with 431 patients newly diagnosed with CAD and 139 individuals without CAD (Table 1). The Mean patient age was 51 years (IQR, 43–54). There were 484 men (84.9%). Within these three groups, statistically significant differences were observed in age, sex, smoking, TC, TG, HDL-C, LDL-C, HbA1c, TyG index, AIP, and glucose metabolism (P < 0.05).

**Table 1:** Clinicodemographic features of the non-CAD and CAD groups

	Non-CAD (n = 139)	Single-vessel CAD (n = 144)	Multi-vessel CAD (n = 287)	P value
Age (year)	51 (43, 54)	50 (43, 54)	52 (47, 55)	0.002
Male, n (%)	98 (70.5)	121 (84.0)	265 (92.3)	< 0.001
SBP (mmHg)	130 (122, 148)	129.5 (119, 144)	134 (120, 150)	0.220
DBP (mmHg)	85 (73, 95)	85 (75, 93)	86 (76, 97)	0.206
BMI (kg/m^2^)	24.91 (22.86, 27.01)	24.68 (22.93, 27.19)	25.06 (23.34, 27.34)	0.557
Smoking status				
Current, n (%)	41 (29.50)	80 (55.56)	195 (67.94)	< 0.001
Former, n (%)	13 (9.35)	5 (3.47)	14 (4.88)	0.074
Never, n (%)	85 (61.15)	59 (40.97)	78 (27.18)	< 0.001
Drinking status				
Current, n (%)	25 (17.98)	34 (23.61)	58 (20.21)	0.495
Former, n (%)	5 (3.60)	2 (1.39)	7 (2.44)	0.487
Never, n (%)	109 (78.42)	108 (75.00)	222 (77.35)	0.778
TC (mmol/L)	4.42 (3.89, 5.12)	4.82 (4.08, 5.49)	4.83 (4.18, 5.67)	0.001
TG (mmol/L)	1.4 (1.05, 2.18)	1.75 (1.21, 2.80)	1.84 (1.38, 2.82)	< 0.001
HDL-C (mmol/L)	1.16 (0.90, 1.37)	1.02 (0.88, 1.18)	0.91 (0.80, 1.09)	< 0.001
LDL-C (mmol/L)	2.97 (2.24, 3.52)	3.20 (2.48, 3.81)	3.27 (2.73, 4.03)	< 0.001
HbA1c (%)	5.8 (5.6, 6.0)	5.9 (5.6, 6.6)	6.0 (5.7, 6.7)	< 0.001
TyG index	2.93 (2.63, 3.41)	3.31 (2.87, 3.78)	3.39 (3.03, 3.83)	< 0.001
AIP	0.09 (0, 0.37)	0.24 (0.04, 0.47)	0.31 (0.11, 0.51)	< 0.001
Hypertension, n (%)	53 (38.13)	55 (38.19)	136 (47.39)	0.084
Glucose metabolic states				
NGR (n, %)	93 (66.91)	64 (44.44)	99 (34.49)	< 0.001
Pre-DM (n, %)	31 (22.30)	41 (28.47)	114 (39.72)	0.001
DM (n, %)	15 (10.79)	38 (26.39)	75 (26.13)	0.001
Status of antihypertensive drugs				
Current, n (%)	33 (23.74)	34 (23.61)	77 (26.83)	0.687
Former, n (%)	5 (3.60)	9 (6.25)	28 (9.76)	0.075
Never, n (%)	101 (72.66)	101 (70.14)	182 (63.41)	0.131
Status of antidiabetic drugs				
Current, n (%)	7 (5.04)	14 (9.72)	34 (11.85)	0.083
Former, n (%)	0	1 (0.69)	5 (1.74)	0.228
Never, n (%)	132 (94.96)	129 (89.58)	248 (86.41)	0.028
Status of antilipidemic drugs				
Current, n (%)	33 (23.74)	29 (20.14)	51 (17.77)	0.348
Former, n (%)	2 (1.44)	4 (2.78)	7 (2.44)	0.729
Never, n (%)	104 (74.82)	111 (77.08)	229 (79.79)	0.493
Status of antiplatelet drugs				
Current, n (%)	26 (18.71)	31 (21.53)	49 (17.07)	0.534
Former, n (%)	1 (0.72)	2 (1.39)	7 (2.44)	0.416
Never, n (%)	112 (80.57)	111 (77.08)	231 (80.49)	0.677

*CAD* coronary artery disease, *SBP* systolic blood pressure, *DBP* diastolic blood pressure, *BMI* body mass index, *TG* triglyceride, *TC* total cholesterol, *HDL-C* high-density lipoprotein cholesterol, *LDL-C* low-density lipoprotein cholesterol, *HbA1c* glycosylated hemoglobin, *TyG index* triglyceride-glucose index, *AIP* atherogenic index of plasma, *NGR* normoglycemia, *Pre-DM* pre-diabetes mellitus, *DM* diabetes mellitus

### Associations of the TyG index and AIP with CAD

The TyG index was distributed across four groups based on quartiles: I (0 ≤ TyG < 2.86), II (2.86 ≤ TyG < 3.29), III (3.29 ≤ TyG < 3.76), and IV (3.76 ≤ TyG ≤ 7.08). Logistic regression models unveiled a prominent linkage of the TyG index with CAD, after adjusting for traditional cardiovascular risk elements and medical treatments (P < 0.001, Table 2). With the TyG index as a continuous variate, a notable connection with CAD was demonstrated (OR = 2.317, 95%CI 1.499–3.582; P < 0.001). In addition, with the TyG index as a categorical variate, CAD risk levels were 5.196 (95%CI 2.386–11.312, P < 0.001) fold higher in patients categorized as IV cases versus category I cases after adjustment for confounders.

**Table 2:** Association between the TyG index and CAD

Variables	Coronary artery disease					
	Model 1		Model 2		Model 3	
	OR (95% CI)	P value	OR (95% CI)	P value	OR (95% CI)	P value
TyG index	2.758 (1.975–3.850)	< 0.001	2.770 (1.966–3.903)	< 0.001	2.317 (1.499–3.582)	< 0.001
I	Reference		Reference		Reference	
II	2.891 (1.731–4.827)	< 0.001	2.756 (1.607–4.725)	< 0.001	2.275 (1.234–4.193)	0.008
III	3.718 (2.171–6.366)	< 0.001	3.497 (1.992–6.139)	< 0.001	2.766 (1.429–5.352)	0.003
IV	6.562 (3.546–12.145)	< 0.001	6.774 (3.573–12.842)	< 0.001	5.196 (2.386–11.312)	< 0.001
P-trend	< 0.001		< 0.001		< 0.001	

Model 1: unadjusted

Model 2: adjusted for age and sex

Model 3: adjusted for age, sex, SBP, BMI, smoking status, drinking status, HDL-C, LDL-C, hypertension, status of antihypertensive drugs, status of antidiabetic drugs, status of antilipidemic drugs, status of antiplatelet drugs

*TyG index* triglyceride-glucose index, *CAD* coronary artery disease, *SBP* systolic blood pressure, *BMI* body mass index, *HDL-C* high-density lipoprotein cholesterol, *LDL-C* low-density lipoprotein cholesterol

Likewise, AIP was stratified into four tiers based on quartiles: I (0 ≤ AIP ≤ 0.04), II (0.05 ≤ AIP ≤ 0.24), III (0.25 ≤ AIP ≤ 0.46), and IV (0.47 ≤ AIP ≤ 2.14). As shown in Table 3, the results demonstrated a notable correlation between AIP and CAD after multivariate adjustment (P < 0.05). With AIP as a continuous variable, a significant correlation with CAD was firmly established (OR = 3.897, 95%CI 1.489–10.198, P < 0.05). With AIP as a categorical variate, CAD risk was 2.425-fold higher in cases categorized as IV compared with category I cases (95%CI 1.201–4.898, P < 0.05).

**Table 3:** Association between AIP and CAD

Variables	Coronary artery disease					
	Model 1		Model 2		Model 3	
	OR (95% CI)	P value	OR (95% CI)	P value	OR (95% CI)	P value
AIP	5.981 (2.628–13.608)	< 0.001	5.210 (2.257–12.027)	< 0.001	3.897 (1.489–10.198)	0.006
I	Reference		Reference		Reference	
II	1.854 (1.123–3.059)	0.016	1.719 (1.014–2.913)	0.044	1.326 (0.722–2.435)	0.363
III	2.920 (1.691–5.042)	< 0.001	2.599 (1.471–4.591)	0.001	2.046 (1.063–3.937)	0.032
IV	3.506 (1.978–6.213)	< 0.001	3.218 (1.775–5.835)	< 0.001	2.425 (1.201–4.898)	0.014
P-trend	< 0.001		< 0.001		0.007	

Model 1: unadjusted

Model 2: adjusted for age and sex

Model 3: adjusted for age, sex, SBP, BMI, smoking status, drinking status, LDL-C, HbA1c, DM, hypertension, status of antihypertensive drugs, status of antidiabetic drugs, status of antilipidemic drugs, status of antiplatelet drugs

*AIP* atherogenic index of plasma, *CAD* coronary artery disease, *SBP* systolic blood pressure, *BMI* body mass index, *LDL-C* low-density lipoprotein cholesterol, *HbA1c* glycosylated hemoglobin, *DM* diabetes mellitus

### Associations of the TyG index and AIP with CAD severity

Logistic regression models revealed that the TyG index displayed no statistical significance with CAD severity (P > 0.05, Table 4). Notably, the results demonstrated a significant link between AIP and CAD severity (P < 0.05, Table 5).

**Table 4:** Association between the TyG index and CAD severity

Variables	Multi-vessel coronary artery disease					
	Model 1		Model 2		Model 3	
	OR (95% CI)	P value	OR (95% CI)	P value	OR (95% CI)	P value
TyG index	1.292 (0.964–1.723)	0.086	1.403 (1.035–1.903)	0.029	1.177 (0.822–1.686)	0.374
T1	Reference		Reference		Reference	
T2	1.745 (1.002–3.038)	0.49	1.563 (0.884–2.764)	0.125	1.533 (0.826–2.843)	0.176
T3	2.019 (1.139–3.579)	0.016	1.913 (1.072–3.475)	0.028	1.679 (0.884–3.190)	0.113
T4	1.654 (0.948–2.888)	0.077	1.840 (1.032–3.283)	0.039	1.449 (0.727–2.885)	0.292
P-trend	0.085		0.036		0.312	

Model 1: unadjusted

Model 2: adjusted for age and sex

Model 3: adjusted for age, sex, SBP, BMI, smoking status, drinking status, HDL-C, LDL-C, hypertension, status of antihypertensive drugs, status of antidiabetic drugs, status of antilipidemic drugs, status of antiplatelet drugs

*TyG index* triglyceride-glucose index, *CAD* coronary artery disease, *SBP* systolic blood pressure, *BMI* body mass index, *HDL-C* high-density lipoprotein cholesterol, *LDL-C* low-density lipoprotein cholesterol

**Table 5:** Association between AIP and CAD severity

Variables	Multi-vessel coronary artery disease					
	Model 1		Model 2		Model 3	
	OR (95% CI)	P value	OR (95% CI)	P value	OR (95% CI)	P value
AIP	1.954 (0.961–3.973)	0.064	2.279 (1.071–4.846)	0.032	2.386 (1.026–5.548)	0.043
T1	Reference		Reference		Reference	
T2	1.777 (1.012–3.120)	0.045	1.611 (0.902–2.876)	0.107	1.759 (0.953–3.249)	0.071
T3	1.577 (0.906–2.745)	0.107	1.495 (0.837–2.671)	0.174	1.700 (0.908–3.182)	0.097
T4	1.721 (0.984–3.012)	0.057	1.816 (1.009–3.271)	0.047	1.925 (0.998–3.714)	0.051
P-trend	0.095		0.070		0.072	

Model 1: unadjusted

Model 2: adjusted for age and sex

Model 3: adjusted for age, sex, SBP, BMI, smoking status, drinking status, LDL-C, HbA1c, DM and hypertension, status of antihypertensive drugs, status of antidiabetic drugs, status of antilipidemic drugs, status of antiplatelet drugs

*AIP* atherogenic index of plasma, *CAD* coronary artery disease, *SBP* systolic blood pressure, *BMI* body mass index, *LDL-C* low-density lipoprotein cholesterol, *HbA1c* glycosylated hemoglobin, *DM* diabetes mellitus

### Associations of the TyG index and AIP with CAD severity in different glucose metabolic states

As shown in Table 6, the TyG index exhibited no statistical significance with CAD severity irrespective of the glucose metabolism  states (P > 0.05). However, AIP in NGR patients exhibited a discernible link with CAD severity (P < 0.05, Table 7).

**Table 6:** Association between the TyG index and CAD severity in different glucose metabolic states

Glucose metabolic state	Multi-vessel coronary artery disease					
	Model 1		Model 2		Model 3	
	OR (95% CI)	P value	OR (95% CI)	P value	OR (95% CI)	P value
NGR						
TyG index	1.764 (0.944–3.295)	0.075	1.873 (0.970–3.617)	0.062	2.105 (0.906–4.893)	0.084
T1	Reference		Reference		Reference	
T2	1.345 (0.562–3.217)	0.506	1.334 (0.522–3.409)	0.547	1.665 (0.571–4.859)	0.350
T3	1.548 (0.646–3.707)	0.327	1.397 (0.559–3.490)	0.474	1.653 (0.529–5.160)	0.387
T4	2.222 (0.892–5.534)	0.086	2.515 (0.948–6.670)	0.064	3.215 (0.943–10.967)	0.062
P-trend	0.083		0.066		0.073	
Pre-DM						
TyG index	1.231 (0.672–2.254)	0.502	1.279 (0.680–2.406)	0.445	0.741 (0.345–1.591)	0.442
T1	Reference		Reference		Reference	
T2	2.095 (0.725–6.059)	0.172	2.165 (0.739–6.348)	0.159	2.286 (0.602–8.677)	0.225
T3	1.481 (0.540–4.062)	0.445	1.449 (0.520–4.035)	0.478	0.789 (0.221–2.815)	0.715
T4	0.821 (0.315–2.139)	0.686	0.856 (0.313–2.341)	0.761	0.347 (0.098–1.231)	0.101
P-trend	0.481		0.124		0.048	
DM						
TyG index	1.007 (0.603–1.682)	0.977	1.190 (0.661–2.144)	0.562	1.036 (0.948–1.132)	0.430
T1	Reference		Reference		Reference	
T2	0.947 (0.319–2.812)	0.922	0.958 (0.311–2.951)	0.940	0.871 (0.201–3.766)	0.853
T3	1.447 (0.475–4.410)	0.515	1.940 (0.593–6.345)	0.273	2.603 (0.581–11.676)	0.211
T4	0.842 (0.280–2.530)	0.760	1.166 (0.344–3.955)	0.805	0.951 (0.192–4.700)	0.951
P-trend	0.904		0.627		0.857	

Model 1: unadjusted

Model 2: adjusted for age and sex

Model 3: adjusted for age, sex, SBP, BMI, smoking status, drinking status, HDL-C, LDL-C, hypertension, status of antihypertensive drugs, status of antidiabetic drugs, status of antilipidemic drugs, status of antiplatelet drugs

*TyG index* triglyceride-glucose index, *CAD* coronary artery disease, *SBP* systolic blood pressure, *BMI* body mass index, *HDL-C* high-density lipoprotein cholesterol, *LDL-C* low-density lipoprotein cholesterol

**Table 7:** Association between AIP and CAD severity in different glucose metabolic states

Glucose metabolic state	Multi-vessel coronary artery disease					
	Model 1		Model 2		Model 3	
	OR (95% CI)	P value	OR (95% CI)	P value	OR (95% CI)	P value
NGR						
AIP	3.842 (1.032–14.302)	0.045	4.199 (1.045–16.881)	0.043	5.568 (1.099–28.200)	0.038
I	Reference		Reference		Reference	
II	1.283 (0.539–3.056)	0.573	1.202 (0.480–3.009)	0.694	2.427 (0.835–7.052)	0.103
III	1.477 (0.620–3.521)	0.379	1.516 (0.603–3.811)	0.376	1.758 (0.570–5.416)	0.326
IV	2.045 (0.823–5.084)	0.123	2.093 (0.799–5.479)	0.133	3.524 (1.140–10.896)	0.029
P-trend	0.121		0.115		0.063	
Pre-DM						
AIP	1.513 (0.432–5.306)	0.518	1.565 (0.413–5.939)	0.510	0.950 (0.205–4.407)	0.948
I	Reference		Reference		Reference	
II	0.931 (0.347–2.497)	0.887	0.936 (0.342–2.560)	0.897	0.812 (0.247–2.664)	0.731
III	1.470 (0.518–4.167)	0.469	1.413 (0.487–4.098)	0.524	1.235 (0.346–4.405)	0.745
IV	0.931 (0.347–2.497)	0.887	0.928 (0.332–2.597)	0.887	0.437 (0.121–1.584)	0.208
P-trend	0.972		0.955		0.237	
DM						
AIP	1.096 (0.331–3.633)	0.881	1.324 (0.346–5.060)	0.682	0.907 (0.177–4.650)	0.907
I	Reference		Reference		Reference	
II	1.625 (0.543–4.865)	0.386	1.457 (0.469–4.522)	0.515	1.067 (0.250–4.548)	0.930
III	3.611 (1.092–11.944)	0.035	3.353 (0.951–11.816)	0.060	2.774 (0.548–14.029)	0.217
IV	0.843 (0.295–2.409)	0.749	0.928 (0.286–3.008)	0.901	0.550 (0.115–2.623)	0.453
P-trend	0.978		0.903		0.459	

Model 1: unadjusted

Model 2: adjusted for age and sex

Model 3: adjusted for age, sex, SBP, BMI, smoking status, drinking status, LDL-C, hypertension, status of antihypertensive drugs, status of antidiabetic drugs, status of antilipidemic drugs, status of antiplatelet drugs

*AIP* atherogenic index of plasma, *CAD* coronary artery disease, *SBP* systolic blood pressure, *BMI* body mass index, *LDL-C* low-density lipoprotein cholesterol

### Predictive values of the TyG index and AIP in CAD

**Fig. 2:**
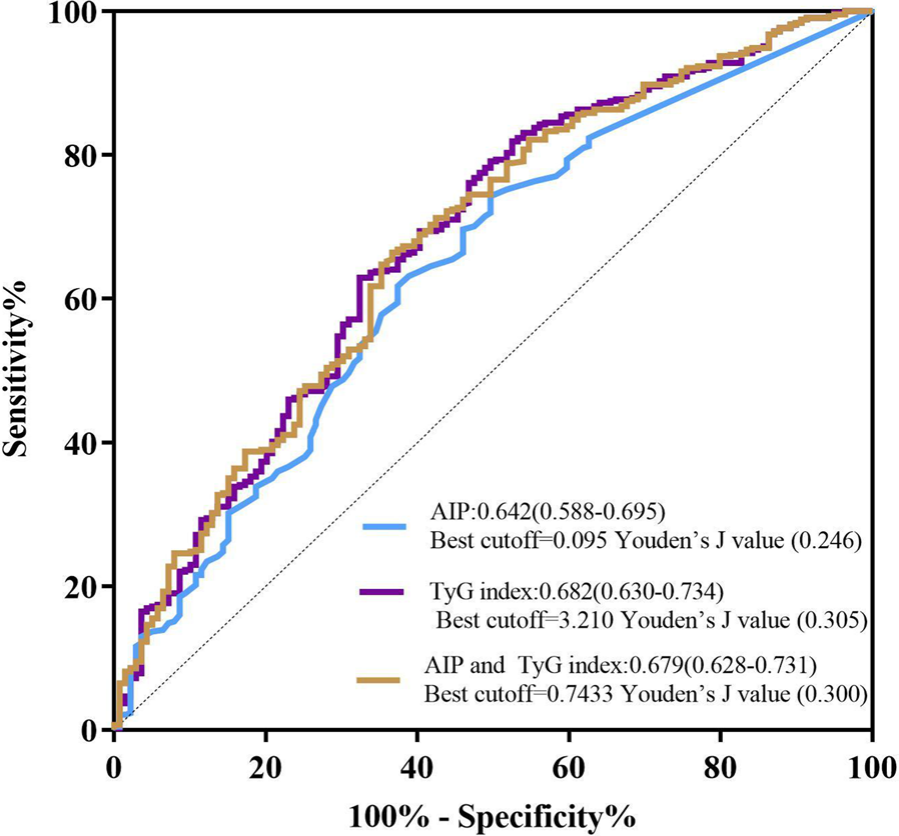
ROC curve analysis of the TyG index and AIP for CAD prediction. *ROC* receiver operating characteristic, *TyG* triglyceride-glucose, *AIP* atherogenic index of plasma, *CAD* coronary artery disease

The ROC curve analysis of the TyG index and AIP for CAD prediction is shown in Fig. 2. The AUCs of the TyG index and AIP for predicting CAD were 0.682 and 0.642 (both P < 0.001), respectively, and their optimal cut-off values were 3.210 (Youden index: 0.305) and 0.095 (Youden index: 0.246), respectively.

## Discussion

The present study first assessed the associations of the TyG index and AIP with new-onset CAD severity in different states of glucose metabolism. The principal findings were: (1) The TyG index was not associated with the severity of new-onset CAD, regardless of glucose metabolic states; (2) AIP had a notable correlation with multi-vessel CAD in the NGR population; and (3) The TyG index and AIP were promising biomarkers for predicting CAD newly diagnosed, displaying similar predictive performance.

IR is a major feature of T2DM and an important risk factor for cardiovascular disease (CVD) [5, 6]. IR is susceptible to inflammation, oxidative stress, vascular endothelial dysfunction, and multiple metabolic disorders, which may be the primary mechanisms for CVD occurrence and progression [7]. IR not only promotes the occurrence of CVD in the general population and T2DM patients but also can predict the outcomes of CVD [18, 19]. In addition, IR may increase the risk of frailty, muscle loss, and cognitive impairment [20, 21].

Accurate identification of IR is expected to establish more effective cardiovascular risk stratification to improve cardiovascular primary and secondary prevention. The Hyperinsulinemic euglycemic clamp test is considered the ultimate tool for IR, but its high cost, complexity, laboriousness, and time consumption limit its application in clinical practice [22]. Homeostasis model assessment for IR (HOMA-IR) is a means for evaluating IR [21], but has limited value for subjects receiving insulin treatment or patients with severely impaired beta-cell function [23]. To address this limitation, the TyG index has been developed, which is superior to HOMA-IR in evaluating IR [24, 25]. Mounting evidence  reveals that TyG-based indexes are associated with diseases related to chronic inflammation or metabolic disorders, including arterial stiffness [26], albuminuria [27], nonalcoholic fatty liver disease [28, 29], hearing impairment [30], and testosterone deficiency [31]. A recent finding reported that the TyG index had a linkage to CVD in patients with ischemic heart failure after percutaneous coronary intervention [32]. The TyG index was confirmed to be independently related to MACE in individuals with T2DM and acute coronary syndrome [18]. Moreover, a study revealed a correlation between the TyG index and the risk of prehypertension or hypertension in the Japanese population with NGR [33]. Therefore, the TyG index exhibited a substantial link with CVD risk [6, 19, 34, 35]. The adverse association was mainly mediated by the increased prevalence of T2DM, dyslipidemia and hypertension [19].

The relationship between the TyG index and CAD severity in various states of glucose metabolism has been controversial. A retrospective multi-center study encompassing 731 CAD patients reported that the TyG index  exhibited an association with multi-vessel CAD in the T2DM population but not in the pre-DM or NGR groups [36]. On the contrary, another retrospective study involving 2792 CAD patients showed that the TyG index had a correlation with multi-vessel CAD in prediabetic patients, while displaying no such association in T2DM or NGR population [2]. The selection of enrolled patients may contribute to these conflicting results. In these studies, the potential effects of long-term use of secondary prevention drugs for CAD, antidiabetic drugs, and the development of healthier lifestyle habits  could not be excluded. Therefore, we selected new-onset CAD patients as our study object to avoid the prevalence-incidence bias as far as possible. Consistent with the previous study [37], the present study confirmed the TyG index  was a risk factor for new-onset CAD. In addition, our study demonstrated that the TyG index was not correlated with new-onset CAD severity, regardless of glucose metabolic states.

Dyslipidemia is a traditional cardiovascular risk factor as well as a key driver for the occurrence and progression of coronary atherosclerosis [38]. Considering the complex interaction of lipoprotein metabolism, compared with a single blood lipid, AIP based on the combination of TG and HDL-C is considered to be a useful marker to reflect atherosclerosis [39]. AIP has been found to exhibit a correlation with CVD risk [40]. In addition, AIP has been found to be correlated with CAD severity based on the Synergy Between Percutaneous Coronary Intervention with Taxus and Cardiac Surgery (SYNTAX) score assessment [41]. Notably, the SYNTAX score  cannot directly reflect the number of coronary lesions [41]. AIP has been reported to be significantly associated with T2DM [12, 13]. However, the relationship between AIP and the severity of new-onset CAD in different glucose metabolic states has not been clarified. Consistent with previous findings [42], our study confirmed that AIP was a risk element for CAD. Furthermore, our study first found that AIP in NGR patients had a notable connection with new-onset CAD severity, but there  was no such correlation in pre-DM and T2DM populations. This finding suggests that IR may interfere with the relationship between AIP and CAD severity. However, more studies are needed to validate our results and explore the mechanisms in the future.

A recent finding demonstrated that the TyG index and AIP could predict subclinical CAD [43]. However, there is currently no study specifically comparing the predictive values of these two indices for newly diagnosed CAD. Our study first found that these two indexes had similar predictive performance for CAD. The TyG index and AIP are expected to serve as simple and applicable biomarkers to recognize individuals at high risk of CAD early, achieving more targeted treatment or prevention.

## Strengths and limitations

The strength of this study is that it is the first study to specifically evaluate the effects of AIP and the TyG index on new-onset CAD severity under different glucose metabolic statuses. All CAD patients were those who underwent first-time CAG and were newly diagnosed with CAD, which was beneficial to avoid potential impacts of long-term use of secondary prevention drugs for CAD and healthier lifestyle habits and to avoid the prevalence-incidence bias.

There are also some limitations to this study. Firstly, the TyG index and AIP  were determined based on baseline data, which could not evaluate their longitudinal associations with CVD risk over time. Having dynamic data may add value to the risk stratification of CAD. Secondly, the relatively small size of the patient cohort might reduce the statistical robustness of the findings. Thirdly, the potential impact of the long-term use of antihypertensive, antidiabetic, and antilipidemic drugs on the measurement of blood lipids and glucose levels as well as CAD occurrence cannot be excluded. Fourthly, there is a lack of data on dietary habits, as diet may be an important confounding factor. Fifthly, as a retrospective observational study, the present study was unable to establish causality and could not completely exclude residual confounding effects, such as activity habits, although many confounding factors were adjusted. Sixthly, this single-center study involving the Chinese population might lead to admission rate bias, and the findings  might not apply to broader populations. Further prospective, large-scale, multi-center randomized controlled trials may make our conclusions more reliable. Future studies should consider these factors to further improve the accuracy and validity of the results.

## Conclusion

The TyG index and AIP were closely related to newly diagnosed CAD as well as having similar predictive values for CAD. These two indices could be widely used in clinical practice to identify high-risk CAD populations early. The TyG index had no association with new-onset CAD severity, regardless of glucose metabolic states. AIP exhibited an independent link with the severity of new-onset CAD in NGR patients, but not in the pre-DM or T2DM populations. Monitoring AIP is expected to discover NGR individuals at high risk of severe and complex CAD early, providing novel prevention strategy for the clinical management.

## Data Availability

Due to privacy and ethical limitations, the data generated and analyzed in the current study are not publicly available but can be obtained from corresponding authors upon reasonable request.

## Abbreviations

TyG: Triglyceride-glucose
AIP: Atherogenic index of plasma
CAD: Coronary artery disease
NGR: Normal glucose regulation
Pre-DM: Prediabetes mellitus
DM: Diabetes mellitus
ROC: Receiver operating characteristic
AUC: Area under the curve
CAG: Coronary angiography
T2DM: Type 2 diabetes mellitus
IR: Insulin resistance
FPG: Fasting plasma glucose
TG: Triglyceride
HDL-C: High-density lipoprotein cholesterol
BMI: Body mass index
TC: Total cholesterol
LDL-C: Low-density lipoprotein cholesterol
HbA1c: Glycosylated hemoglobin
IQR: Interquartile range
OR: Odds ratio
CI: Confidence interval
SBP: Systolic blood pressure
CVD: Cardiovascular disease
HOMA-IR: Homeostasis model assessment for IR
SYNTAX: Synergy Between Percutaneous Coronary Intervention with Taxus and Cardiac Surgery
